# Autoantibodies in Systemic Vasculitis

**DOI:** 10.3389/fimmu.2015.00184

**Published:** 2015-04-22

**Authors:** Alexandre Wagner Silva de Souza

**Affiliations:** ^1^Rheumatology Division, Department of Internal Medicine, Universidade Federal de São Paulo-Escola Paulista de Medicina, São Paulo, Brazil

**Keywords:** autoantibodies, systemic vasculitis, ANCA, cryoglobulinemia, anti-GBM antibodies

## Introduction

Systemic vasculitis is a heterogeneous group of disorders characterized by inflammation and necrosis in the vessel wall. The diagnosis of a systemic vasculitis is challenging, because patients usually present a broad spectrum of manifestations that vary according to the predominant size of vessels affected, organs and systems involved, and the extent of the inflammatory process (1). In systemic vasculitis, disease manifestations usually cluster into clinical phenotypes and definite diagnosis rely on confirmation by tissue biopsy, angiography, or by serologic tests. However, when a systemic vasculitis is under investigation, it is of paramount importance to keep in mind vasculitis mimics (e.g., genetic vascular disorders and atheroembolic diseases) and secondary causes (i.e., infections, malignancy, connective tissue disorders, or drugs) (2).

The main serologic tests for the diagnosis of primary systemic vasculitides are antineutrophil cytoplasmic antibodies (ANCA), cryoglobulins, anti-glomerular basement membrane (anti-GBM) antibodies, and anti-C1q antibodies (3). Although, several other autoantibodies have been investigated in systemic vasculitis, the clinical usefulness of these antibodies still needs further investigation (1, 2) (Table 1). Herein, the main autoantibodies and their clinical associations in systemic vasculitis are reviewed.

**Table 1:** **Autoantibodies in primary systemic vasculitides**.

Vasculitis	Autoantibodies
Takayasu arteritis	AECA
	Anti-aorta antibodies
	Anti-ferritin antibodies
	Anti-annexin V antibodies
	Anti-monocyte antibodies
Giant cell arteritis	AECA
	Anti-ferritin antibodies
Polyarteritis nodosa	AECA
Kawasaki disease	AECA
ANCA-associated vasculitis	AECA
	PR3-ANCA
	MPO-ANCA
	Anti-LAMP-2 antibodies
Immune complex vasculitis	Anti-C1q antibodies
	Anti-GBM antibodies
	Cryoglobulins
	IgA-ANCA
	Rheumatoid factor

*AECA, antiendothelial cell antibodies; ANCA, antineutrophil cytoplasmic antibodies; GBM, glomerular basement membrane; LAMP-2, lysosome associated membrane protein-2; MPO, myeloperoxidase; PR3, proteinase 3*.

## Antineutrophil Cytoplasmic Antibodies

Antineutrophil cytoplasmic antibodies are the diagnostic biomarkers for some small-vessel necrotizing vasculitis such as granulomatosis with polyangiitis (GPA) (formerly Wegener’s granulomatosis), mycroscopic polyangiitis (MPA), eosinophilic granulomatosis with polyangiitis (EGPA) (formerly Churg–Strauss syndrome), and renal limited vasculitis (RLV). The main target antigens of ANCA are proteinase 3 (PR3) and myeloperoxidase (MPO) located on primary granules of neutrophils and lysosomes of monocytes. ANCA reactivity is variable and differences in affinity of ANCA with PR3 antigen have been described with a higher inhibition of PR3 activity observed in GPA patients in remission compared with those with active disease. Unlike, PR3-ANCA, myeloperoxidase-antineutrophil cytoplasmic antibodies (MPO-ANCA) recognize a restricted number of epitopes in MPO and do not impair its enzymatic activity. When ANCA bind to their target antigen, they activate neutrophils and induce the release of granule enzymes, pro-inflammatory cytokines, and the generation of respiratory burst, leading to endothelial damage and eventually to the vasculitic process (4).

Currently, the main assays for the detection of ANCA in sera are indirect immunofluorescence (IIF) on ethanol-fixed human neutrophils and enzyme-linked immunosorbent assay (ELISA) for antigen specificity. The International Consensus Statement on Testing and Reporting ANCA recommends the combination of both techniques (IIF and ELISA) for the detection of ANCA in patients under investigation for ANCA-associated vasculitis, since 10% of ANCA positive GPA or MPA patients present only IIF positive results (5). The main patterns observed in IIF are the cytoplasmic (C-ANCA) pattern that displays a diffuse granular cytoplasmic staining with interlobular accentuation and the perinuclear (P-ANCA) pattern that shows perinuclear fluorescence with nuclear extension. C-ANCA is mostly associated with anti-PR3 antibodies, whereas P-ANCA is associated with anti-MPO antibodies. Another ANCA pattern described is the atypical ANCA, also referred as A-ANCA. A-ANCA usually shows perinuclear staining without nuclear extension, or diffuse flat cytoplasmic staining, or the combination of both cytoplasmic and nuclear/perinuclear staining on neutrophils (5, 6).

Antineutrophil cytoplasmic antibodies are positive in up to 90% of patients with active generalized GPA, while only 40% of patients with localized disease present a positive ANCA test. PR3-ANCA is found in up to 90% of GPA patients with a positive ANCA. The presence of MPO-ANCA is observed in MPA, EGPA, and with RLV with a rate of positivity of 70, 50, and 80%, respectively. The combination of IIF and ELISA for PR3-ANCA or MPO-ANCA yields a specificity of 99% (4). ANCA specificity in ANCA-associated vasculitis is more closely related to disease phenotype rather than clinical diagnosis. Patients presenting renal involvement and small-vessel vasculitis without evidence of granulomatous inflammation are more likely to have MPO-ANCA, whereas PR3-ANCA is more associated with necrotizing granulomatous inflammation and more frequent relapses compared with MPO-ANCA. Moreover, results from a genome wide association study that evaluated HLA and SERPINA1 genes showed that genetic background in ANCA-associated vasculitis is related with ANCA-specificity rather than with disease phenotype. ANCA levels often fluctuate following disease activity, but they are not meant to guide therapeutic decisions (7).

Myeloperoxidase-antineutrophil cytoplasmic antibodies are also found in 30% of patients with anti-GBM disease (4). A-ANCA is associated with antibodies against other target antigens in cytoplasmic granules of neutrophils such as lactoferrin, lysozyme, azurocidin, elastase, cathepsin G, and bactericidal/permeability increasing enzyme (BPI) (8). A-ANCA may be found in inflammatory bowel disease, mostly ulcerative colitis, autoimmune hepatitis, primary sclerosing cholangitis, rheumatoid arthritis, and drug exposure or drug-induced ANCA-associated vasculitis (6, 8).

During the last few years, different immunoassays have been developed for the detection of ANCA including second and third generation ELISA tests, automated systems for analyzing fluorescence patterns, chemiluminescent immunoassays, lateral flow assay systems, and bead-based multiplex assays (6–8). The first generation ELISA used to detect PR3-ANCA and MPO-ANCA applies absorption coating methods with target antigens directly immobilized to the surface of the plate what induces masking and deformation of epitopes. This direct ELISA has significant variation amongst kits and lacks sensitivity (6, 7). Second and third generation ELISA tests were developed to improve sensitivity. The second generation ELISA is also referred as capture ELISA and uses capture molecules, mostly monoclonal antibodies to bind the target antigen to the plate surface, while the third generation ELISA uses anchor molecules to immobilize the antigen to the plate surface. A study that compared three generations of PR3-ANCA assays found the highest sensitivity with the third generation anchor ELISA: 96.0% [95% confidence interval (CI): 79.6–99.3%], followed by IIF: 92.0% (95% CI: 73.9–98.8%), by second generation capture ELISA: 72.0% (95% CI: 50.6–87.9%), and by first generation direct ELISA: 60.0% (95% CI: 38.7–78.8%) (9).

Despite advances in assays to detect ANCA, to date the combination of IIF with the ELISA test to detect ANCA specificity is still recommended, preferably with second or third generation ELISA. Although, chemiluminescent immunoassay has similar or superior diagnosis performance for ANCA-associated vasculitis compared to conventional assays, the role of novel techniques to detect ANCA deserves further studies (7, 8).

## Anti-Lysosome Associated Membrane Protein-2 Antibodies

The lysosome associated membrane protein-2 (LAMP-2) is a heavily glycosilated protein present in lysosomes, but it also traffics to the cell surface where it is expressed by monocytes, neutrophils, and endothelial cells. Moreover, LAMP-2 may be also found within the membrane of MPO/PR3 intracellular vesicles of neutrophils. Thus, anti-LAMP-2 antibodies are considered a subtype of ANCA. The pathogenicity of anti-LAMP-2 antibodies is highlighted by the striking 100% homology observed between the epitope P_41–49_ of LAMP-2 and a sequence in FimH, a bacterial adhesin present in fimbriated bacterial such as *Escherichia coli* (10).

Anti-LAMP-2 antibodies were firstly described in 87% of patients with RLV and this high prevalence of anti-LAMP-2 antibodies was further confirmed in a multicenter study that included patients with ANCA-associated vasculitis (i.e., GPA, MPA, and RLV) at disease presentation from Austria, the Netherlands, and from the United Kingdom with a positivity of 89%, 91%, and 80%, respectively. In contrast, anti-LAMP-2 antibodies were detected in only 7% of patients in remission. However, the prevalence of anti-LAMP-2 antibodies was only 21% in patients with ANCA-associated vasculitis from a study performed in North Carolina. The reasons for these controversial results are probably due to different methods used to detect anti-LAMP-2 antibodies in both studies and to the inclusion of patients in different phases of the disease (e.g., active and quiescent disease) in the latter study (10).

## Cryoglobulins

Cryoglobulins are typically immunoglobulins that precipitate *in vitro* at temperatures <37°C and solubilize upon re-warming. Cryoglobulinemia refers to the presence of cryoglobulins in patient’s serum. Besides the quantification of circulating cryoglobulins, it is also necessary to analyze the nature of circulating cryoglobulins (11). Brouet classification is the most frequent way of classifying cryoglobulins, this classification relies on the clonality of immunoglobulins and on rheumatoid factor activity. Type I cryoglobulinemia refers to the presence of monoclonal immunoglobulin, including IgM, IgG, IgA, or light chains (e.g., kappa or lambda) without rheumatoid factor activity. Type I cryoglobulinemia is usually associated with hematologic malignancies (e.g., Waldenström macroglobulinemia or multiple myeloma). Type II cryoglobulinemia is typically defined as the presence of polyclonal immunoglobulins associated with a monoclonal IgM with rheumatoid factor activity. Type III cryoglogulinemia is a mixture of polyclonal IgM and IgG. Both type II and III are referred as mixed cryoglobulinemia and are associated with chronic viral infections, mainly hepatitis C virus (HCV) and connective tissue diseases. In the latter group of diseases, cryoglobulins are a mixture of autoantibodies that become antigenic. The distinction between those autoantibodies and autoantigens is rather difficult. No other cause may be found in up to 10% of patients with mixed cryoglobulinemia and those cases are regarded as essential cryoglobulinemia (12).

Two clinical syndromes are recognized as caused by circulating cryoglobulins in serum: the hyperviscosity syndrome and cryoglobulinemic vasculitis. Although most patients with type I cryoglobulinemia are asymptomatic, the hyperviscosity syndrome is the main set of manifestations in this type of cryoglobulinemia and includes Raynaud’s phenomenon, digital ischemia, renal failure, vision blurring, loss of vision, headache, vertigo, nystagmus, deafness, confusion, coma, and other neurological manifestations (12). Cryoglobulinemic vasculitis usually develops in patients with type II and III cryoglobulinemia and is manifested by palpable purpura, livedo reticularis, Raynaud’s phenomenon, arthralgia, myalgias, glomerulonephritis, and peripheral neuropathy. In severe cases, patients present rapidly progressive glomerulonephritis, central nervous system vasculitis and/or pulmonary vasculitis (11).

## Anti-Glomerular Basement Membrane Antibodies

Anti-glomerular basement membrane antibodies are biomarkers for the diagnosis of anti-GBM antibody disease (formerly Goodpasture’s syndrome), which has been recently classified as immune complex small-vessel vasculitis affecting glomerular and/or pulmonary capillaries. Renal involvement is due to crescentic glomerulonephritis and is usually manifested as rapidly progressive glomerulonephritis, whereas typical pulmonary involvement is pulmonary hemorrhage (3).

Anti-glomerular basement membrane antibodies may be detected by direct IF on renal biopsy, where a linear deposition of IgG is observed on glomerular capillaries. Alternatively, when renal biopsy cannot be performed the ELISA test is used to detect circulating anti-GBM antibodies in patients with active disease and its sensitivity ranges from 65 to 100%. ELISA assays that use purified or recombinant alpha-3 chain of collagen IV present the best sensitivity. Antigen specificity may also be confirmed by Western blot. IIF is rarely performed (13).

## Anti-C1q Antibodies

Anti-C1q antibodies are biomarkers for the diagnosis of hypocomplementemic urticarial vasculitis (HUV). This entity has recently been classified as immune complex small-vessel vasculitis by 2012 Chapel Hill Consensus Conference. Common features of HUV include glomerulonephritis, cutaneous lesions (e.g., wheals that persist for more than 24 h), arthralgia or arthritis, lung involvement, gastrointestinal vasculitis, and ocular inflammation (3).

## Other Autoantibodies

Anti-endothelial cell antibodies (AECA) are a heterogeneous family of antibodies with multiple target antigens on endothelial cell membrane. AECA have been described in several primary systemic vasculitides such as Takayasu arteritis, giant cell arteritis, polyarteritis nodosa, GPA, MPA, EGPA, IgA vasculitis, Kawasaki disease, and Behçet’s disease. However, the usefulness of AECA in clinical practice has still to be determined (14).

Anti-ferritin antibodies are novel IgG autoantibodies against human ferritin heavy chain and its terminal N peptide. Using the combination of results from different ELISA assays, anti-ferritin antibodies have been described in up to 92% of patients with giant cell arteritis/polymyalgia rheumatica and in 62% of Takayasu arteritis patients. However, anti-ferritin antibodies are also found in 28% of patients with systemic lupus erythematosus, in 22% of patients with febrile illnesses, and in 11% of patients with atherosclerotic disease, but not in healthy blood donors (15).

## Concluding Remarks

To date, the investigation of circulating autoantibodies has been shown to be useful for diagnosis of systemic small vessel vasculitis including ANCA-associated vasculitis and immune complex vasculitis. No specific autoantibody has been shown to be of any help in diagnosis or in evaluating disease activity in patients with medium and large vessel vasculitis.

## Conflict of Interest Statement

The author declares that the research was conducted in the absence of any commercial or financial relationships that could be construed as a potential conflict of interest.
